# Genome Analysis of a Historical *Shigella dysenteriae* Serotype 1 Strain Carrying a Conserved Stx Prophage Region

**DOI:** 10.3389/fmicb.2020.614793

**Published:** 2021-01-08

**Authors:** Domonkos Sváb, Linda Falgenhauer, Balázs Horváth, Gergely Maróti, Jane Falgenhauer, Trinad Chakraborty, István Tóth

**Affiliations:** ^1^Institue for Veterinary Medical Research, Centre for Agricultural Research, Martonvásár, Hungary; ^2^Institute of Hygiene and Environmental Medicine, Justus Liebig University Giessen, Giessen, Germany; ^3^German Centre for Infection Research, Site Giessen-Marburg-Langen, Giessen, Germany; ^4^Seqomics Ltd., Mórahalom, Hungary; ^5^Institute of Plant Biology, Biological Research Centre, Szeged, Hungary; ^6^Faculty of Water Sciences, University of Public Service, Baja, Hungary; ^7^Institute for Medical Microbiology, Justus Liebig University Giessen, Giessen, Germany

**Keywords:** *Shigella dysenteriae* serotype 1, Stx1 prophage, complete genome, comparative genomics, phylogeny

## Abstract

*Shigella dysenteriae* are significant agents of bacillary dysentery, accounting for a considerable number of illnesses with high morbidity worldwide. The Shiga toxin (Stx) encoded by a defective prophage is the key virulence factor of *S. dysenteriae* type 1 (SD1) strains. Here we present the full genome sequence of an SD1 strain HNCMB 20080 isolated in 1954, compare it to other sequenced SD1 genomes, and assess the diversity of Stx-prophages harbored by previously sequenced SD1 strains. The genome of HNCMB 20080 consists of a chromosome sized 4,393,622 bp containing 5,183 CDSs, as well as two small plasmids. Comparative genomic analysis revealed a high degree of uniformity among SD1 genomes, including the structure of Stx prophage regions, which we found to form two subgroups termed PT-I and PT-II. All PT-I strains are members of the sequence type (ST) 146 or ST260, while the only PT-II harboring strain, Sd1617 proved to be ST untypeable. In accordance with data from previous reports, the Stx1 prophage could not be induced from HNCMB 20080. Our cumulative data do not support the notion that *stx*-harboring phages in STEC are derived from historical SD1 isolates.

## Introduction

Members of the *Shigella* genus are the leading agents of bacillary dysentery worldwide, especially in developing countries (reviewed by Faherty and Lampel, 2019). Of the four species, *Shigella dysenteriae* is responsible for historical outbreaks of high morbidity and mortality (reviewed by Anderson et al., 2016).

Shiga toxin (Stx) is the main virulence factor of *Shigella dysenateriae* type 1 (SD1) strains first discovered in the late nineteenth century by Kiyoshi Shiga (Trofa et al., 1999). Subsequently, Shiga toxin-production was identified in great variety of *E. coli* (STEC) strains (reviewed by Muniesa and Schmidt, 2014) as well as in other enteral pathogenic bacteria including *Enterobacter cloaceae* (Probert et al., 2014), as well as *S. flexneri* (Gray et al., 2014) and recently *S. sonnei* (Strauch et al., 2001; Carter et al., 2016; Sváb et al., 2017). Two main types of the toxin, Stx1 and Stx2, and several subtypes have been identified in *E. coli*, with Stx1 being almost identical to the one present in *Shigella* strains (Scheutz et al., 2012). Importantly, the *stx* operon is always integrated in the genome of prophages. The Shiga toxin encoding phages constitute a heterogeneous group of temperate lambdoid phages that harbor the *stx* genes among the late region genes (reviewed by Muniesa and Schmidt, 2014).

The Stx prophages carried by non-SD1 strains frequently proved to be inducible and they can spread horizontally, either because, as is the case of many Stx2 prophages, they retained essential genes for the lysis of the host, or in the case of Stx1 prophages, they undergo recombination with Stx2 prophage sequences (Asadulghani et al., 2009; Muniesa and Schmidt, 2014; Tóth et al., 2016). In contrast to these converting Stx phages, the Stx prophages carried by SD1 strains, are cryptic and cannot be induced.

Earlier there was a comprehensive genome sequencing study of a collection of recent and historical SD1 strains producing hundreds of draft genomes (Njamkepo et al., 2016), which shed a light on the evolutionary history of the pathogen, and established an SNP-based phylogeny consisting of four main lineages numbered I–IV. The study, aiming to outline a global phylogeography of SD1 strains, did not venture into details about the history of individual virulence genes or mobile genetic elements.

In the present study, using genome sequencing and by comparative genomic analysis, the complete genome of an antibiotic-sensitive SD1 strain, HNCMB 20080 isolated in Hungary in 1954, was assembled and analyzed, and the Stx prophage region’s great uniformity across currently available SD1 genome sequences was revealed.

## Materials and Methods

### Bacterial Strains and Bacteriophages

*S. dysenteriae* strain HNCMB 20080 was isolated in Hungary from a patient in 1954 and provided by the collection of the Hungarian National Collection of Medical Bacteria (HNCMB), Budapest, Hungary. *E. coli* K-12 MG1655 was used as phage indicator strain. A rifampicin-resistant mutant of HNCMB 20080 was obtained with the method described in Sváb et al. (2018). Briefly, 200 μl of overnight LB culture of HNCMB 20080 was spread onto LB agar plates supplemented with 150 μg/ml rifampicin and incubated at 37°C for 16 h. Single colonies were selected and further purified by several passages on selective plates. One of the resistant colonies was used for further experiments.

### Genome Sequencing, Assembly, and Comparative Analysis

Genomic DNA of SD1 strain HNCMB 20080 was isolated with GenElute Bacterial Genomic DNA Kit (Sigma-Aldrich) according to the manufacturer’s instructions. For genome closure, a combination of short- and long-read sequencing was used. For short-read sequencing, a mate-pair fragment library was generated for the genome of *S. dysenteriae* strain HNCMB 20080 using the Illumina Nextera Mate-Pair Kit (Cat.Num.: FC-132-1001) with insert sizes ranging between 7 and 11 kb. DNA sequencing was carried out on an Illumina MiSeq machine using V2 sequencing chemistry resulting in 2 × 250 nt reads. Long-read sequencing was performed using the Nanopore technology. For this, the library was prepared using the native barcoding kit (EXP-NBD103, Oxford Nanopore Technologies Ltd., Oxford, United Kingdom) combined with 1D chemistry (SQK-LSK108). Sequencing was performed on a SpotON Flow Cell Mk I R9 Version (FLO−MIN106) on a MinION/MinIT machine. Demultiplexing was performed using Porechop v. 0.2.3^1^. Hybrid assembly of short and long reads was performed using Unicycler v. 0.4.7 (Wick et al., 2017) with default parameters. The assembled chromosome and plasmids of HNCMB 20080 are deposited in GenBank under the accession numbers CP061527-CP061529.

Genomic comparisons and searches were performed with megaBLAST available on the NCBI website. Virulence gene search was conducted with VirulenceFinder 2.0.1 (Joensen et al., 2014).

For comparative purposes, assembly of SD1 isolates sequenced by PacBio technologies (Table 1) was performed using ASA^3^P (Schwengers et al., 2020). In order to identify chromosomal inversions in the genome, annotation of all genomes in Table 1 was performed with RASTtk (Brettin et al., 2015), and their alignment was conducted using Progressive Mauve (Darling et al., 2010) through the PATRIC platform (Wattam et al., 2017).

**TABLE 1:** List of complete SD1 genomes available in GenBank as well as draft genomes determined by PacBio sequencing used in this study.

**Strain**	**Alternative name**	**Year of isolation**	**Country**	**Genetic lineage**	**PacBio study/GenBank accession numbers**	**References**	**Stx prophage type**	**MLST type (Achtman)**
CDC ZB4		1991	Zambia	IV	PRJEB7928	Njamkepo et al., 2016	I	146
CDC 69-3818		1969	Guatemala	III	PRJEB7928	Njamkepo et al., 2016	I	146
M115	Crespigny	1926	United Kingdom	I	PRJEB7928	Njamkepo et al., 2016	I	146
Sd197		1950s	China	III	PRJEB7928	Njamkepo et al., 2016	I	146
40-81		1981	India	IV	PRJEB7928	Njamkepo et al., 2016	I	146
CDC 87-3330		1987	Thailand (Ubon)	IV	PRJEB7928	Njamkepo et al., 2016	I	146
CAR10	IPB01016014	2004	Central African Republic (Kanga)	III	PRJEB7928	Njamkepo et al., 2016	I	146
99-9324		1999	Egypt	IV	PRJEB7928	Njamkepo et al., 2016	I	146
17/89		1989	Madagascar	II	PRJEB7928	Njamkepo et al., 2016	I	260
M116	Barsick	1915_1918	United Kingdom (soldier)	II	PRJEB7928	Njamkepo et al., 2016	I	260
Sd1617		1968	Guatemala	Unknown	CP006736	Vongsawan et al., 2015	II	Not typeable*
ATCC 13313		1934	United Kingdom	Unknown	CP026774	Kim et al., 2018	I	260
BU53M1		1990	Burundi	Unknown	CP024466	Kim et al., 2018	I	146
69-3818		Unknown	Unknown	III	CP026777	Kim et al., 2018	I	146
07-3308		Unknown	Unknown	Unknown	CP026878	Kim et al., 2018	I	146
53-3937		Unknown	Unknown	Unknown	CP026780	Kim et al., 2018	I	146
08-3380		Unknown	Unknown	Unknown	CP026782	Kim et al., 2018	I	146
80-547		Unknown	Unknown	Unknown	CP026784	Kim et al., 2018	I	146
NCTC 9718		1955	United Kingdom	Unknown	CP026786	Kim et al., 2018	I	146
Sd197		1950s	China	III	CP000034	Yang et al., 2005	I	146
**HNCMB 20080**	**DRL265/54**	**1954**	**Hungary**	**III**	**CP061527-CP061529**	Njamkepo et al., 2016 **and this study**	**I**	**146**

*^∗^Same as 146, except that the database cannot find the mdh allele. Data regarding strain HNCMB 20080 are highlighted.*

For phylogenetic comparison, core-genome SNP analysis was performed using ParSNP using default parameters (Treangen et al., 2014) and HNCMB 20080 as reference. Core genome sequences were extracted. Recombination events were removed using Gubbins (Croucher et al., 2015; 50 iterations, default parameters) and recombination-diminished SNP counts were determined using the “compute pairwise distance” function of Mega 5.2.2 (default parameters) (Tamura et al., 2011). Based on the pairwise SNP analysis, the phylogenetic relationship of HNCMB 20080 to other Sd1 strains was determined. A tree based on the SNPs differences (neighbor-joining method) was visualized and annotated using Mega 5.2.2.

### Multi-Locus Sequence Typing

Multi-locus sequence typing (MLST) analysis of HNCMB 200080 as well as complete SD1 genomes shown in Table 1 was performed using according to the *E. coli* scheme published by Wirth et al. (2006), version 2.0.4 provided by the Centre for Genetic Epidemiology (Larsen et al., 2012).

### Prophage and Plasmid Search

Prophages sequences present in the genome of HNCMB 20080 were determined using PHASTER with the “precomputed” mode (Arndt et al., 2016). Plasmids were searched for by PlasmidFinder 2.0 (Carattoli et al., 2014).

### Antibiotic Resistance Investigation

Minimum inhibitory concentration (MIC) tests of HNCMB 20080 toward erythromycin were conducted as follows: A liquid culture in LB broth was shaken overnight with 180 rpm at 37°C. Five microliter drops of the culture, containing ~10^5^ cells, were spotted in three parallels on Müller-Hinton agar plates containing concentrations of erythromycin between 1 and 256 μg/ml in halving serial dilution. The lowest concentration of the antibiotic on plates where no growth was observed, was considered the MIC.

### Phage Induction Experiments Prophage Induction

We attempted to mobilize prophages carried by HNCMB 20080 with various agents using standard methods as described previously (Sváb et al., 2013). Briefly, an overnight culture of HNCMB 20080 was diluted 50-fold in LB broth and shaken at 180 rpm for 3 h at 37°C. Afterward, the following induction agents were applied in separate batches: mitomycin C in 0.5 μg/ml, norfloxacin in 1.25 μg/ml final concentration were used (both antibiotics were supplied by Sigma-Aldrich, MO, United States). Phage induction was also attempted by using ultraviolet light, according to the protocol described by Hertman and Luria (1967) with some modifications. Briefly, 1 ml of the 3 h culture was spread in a sterile empty Petri-dish and irradiated with a 30 W UV lamp in a distance of 30 cm for 15 s. In all cases, after the addition of the agent or the irradiation, the cultures were incubated with shaking for another 3 h at 37°C. Following centrifugation at 13,000 g for 1 min, and the supernatant was filtered with a 0.22 μm pore-size sterile filter (Acrodisc Supor, Pall Corporation) and spotted on layered soft agar plates of *E. coli* K-12 derivative strain MG1655. Plates were examined after an overnight incubation at 37°C for the presence of plaques. As control for phage induction, Stx1 and Stx2 phages were induced from non-SD strains (Table 3) by using mitomycin C. *E. coli* K-12 derivative strain MG1655 was used as indicator and propagating strain.

### Attempts for Lysogenization and Phage Transduction

Similar to the phage sensitivity experiments, we attempted to lysogenize *S. dysenteriae* strain HNCMB 20080 with Stx1 and Stx2-carrying phages or their modified derivates. Ten microliter spots of high titer (>10^9^ PFU/ml) Stx1 and Stx2 phage stocks of non-SD1 strains and their 10-fold dilutions were spread on top of layered soft agar plates containing HNCMB 20080. Surviving colonies in the middle of lysis spots were transferred to new agar plates and after overnight incubation at 37°C, they were checked for the presence of *stx* genes by PCR. Phages used in these experiments are listed in Table 3. As an alternative method, in the case of MG1655 *cat*GFP and strain 3538/95, 1 ml of 3 h cultures of the respective strains were mixed with an equal amount of 3 h culture of HNCMB 20080 Rif^R^, and mitomycin C was added in the appropriate concentration. After another 3 h without shaking, 1 ml of the mixed culture was centrifuged and spread onto LB agar plates supplemented with 100 μg/ml rifampicin and 25 μg/ml chloramphenicol, as both MG1655 *cat*GFP and strain 3538/95 contain the *cat* gene encoding chloramphenicol resistance, which trait would also be exhibited by recipient colonies of HNCMB 20080 Rif^R^ in case of successful transduction. After overnight incubation at 37°C, the lactose fermenting ability of the surviving colonies was tested on bromophenol-blue plates, to ensure that only lactose-negative colonies, which are most likely of the recipient SD1 strains and not those of the donor *E. coli* strains, are checked The presence of *stx1* and *stx2* genes was investigated by PCR with the primers and protocol described by Scheutz et al. (2012).

## Results

### Genomic Features

The genome of HNCMB 20080 contains a chromosome and two small plasmids. The chromosome of HNCMB 20080 has a size of 4,393,662 bp. The average GC content is 51.3%, and the chromosome contains 5,183 CDSs and 86 tRNA genes. The first plasmid, pHNCMB20080_1 is 8,953 bp long and contains 13 CDSs. Of these, only two were assigned function, one is replication gene, the other is a household gene. The remaining are all encoding “hypothetical proteins.” The second plasmid, pHNCMB20080_2 is 3,069 bp long and has above 98% homology to *E. coli* strain RHB02-C19 plasmid pRHB02-C19_7 (GenBank CP058069.1). It contains two CDSs, one coding for LPS O-antigen regulator, and the other for an RNA-binding protein. The genomic features of HNCMB 20080 are summarized in Table 2, and the complete list of genes is given in Supplementary Table 1.

**TABLE 2:** List of genomic features of *Shigella dysenteriae* serotype 1 strain HNCMB 20080.

**Genomic element**	**Length (bp)**	**GC%**	**Number of CDSs**	**Virulence genes**	**Antimicrobial resistance genes**	**Prophage regions**	**GenBank accession number**
HNCMB 20080 chromosome	4,393,662	51.3	5,183	*stx1A, stx1B, gad, iroN*	*mdfA*	27 (9 intact)	CP061527
Plasmid pHNCMB20080_1	8,953	39.6	13	–		–	CP061528
Plasmid pHNCMB20080_2	3,069	44.9	2	–		–	CP061529

### Phylogenetic Relations

MLST analysis showed that, similar to the majority of SD1 strains with complete sequence, HNCMB 20080 belongs to ST-146 according to the Achtman scheme (Table 1).

When comparing the HNCMB 20080 genome to the complete genomes in GenBank as well as with nine further PacBio-sequenced genomes which were successfully assembled to enable Progressive Mauve alignment (Table 1), a total of 42 chromosomal inversions were identified ranging in size from around 700 bp to almost 400 kbp (Supplementary Table 2).

**FIGURE 1:**
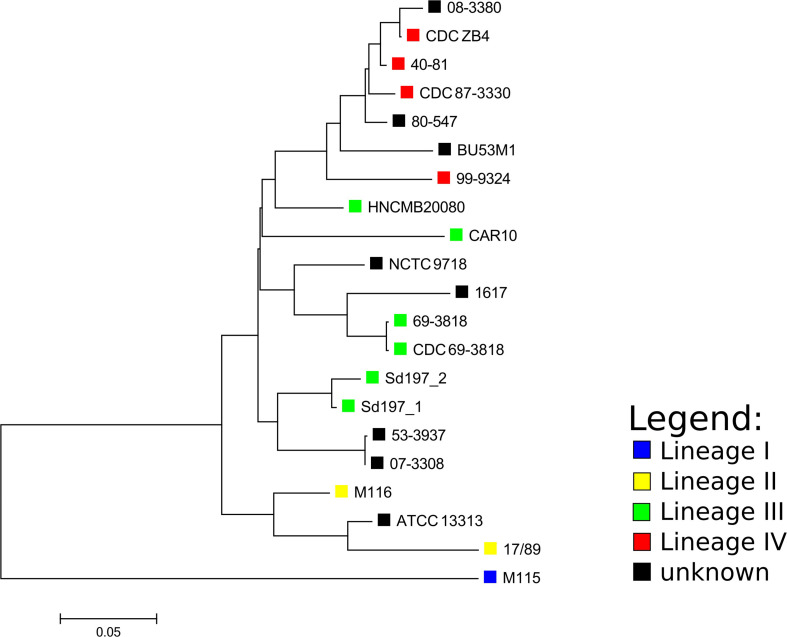
Core SNP-based phylogenetic tree of *Shigella dysenteriae* complete genomes and Pacbio determined assemblies made with ASA^3^P. The SNP analysis was performed with ParSNP, followed by removal of SNPs at recombination sites by Gubbins. The tree was visualized with MEGA. The lineages designated for strains by the SNP-based phylogeny made by Njamkepo et al. (2016) are marked with colored squares where applicable.

Based on the SNP analysis, the closest relatives to HNCMB 20080 were Sd197, 80–547, and 40–81 (Figure 1 and Supplementary Table 3).

### Virulence Profile

Besides the Stx1-encoding gene cluster, VirulenceFinder only identified the *gad* gene encoding glutamate-decarboxylase, and the *iroN* which encodes a *tonB*-dependent outer membrane siderophore receptor protein. The PlasmidFinder search indicated that the pInv plasmid was missing from the strain.

### Prophage Pool

The PHASTER search identified altogether 27 prophage-like regions, with their length between 4.9 and 32 kbp, comprising ~375 kbp of the genome altogether (Supplementary Table 4). Out of these, nine regions were labeled as “intact,” and one of them termed “Region 16” by the program is the Stx1 prophage. The 27 kbp long “Region 15” also contains remnants of a lambdoid prophage, with CDSs encoding a “host killer protein,” CI and cro repressor proteins, and a DNA methylase. The GC content of intact-labeled prophage regions ranged between 48.93 and 52.09%. BLAST searches showed that most of the prophages are present with high coverage and homology in the majority of SD1 strains with complete genomes available, but most of them yielded only low coverage homologies to other enterobacterial phage sequences when BLASTed against viral sequences alone.

### Characteristics of the Stx Encoding Prophage

The prophage region containing the genes encoding the *stx1* operon in HNCM 20080 comprises 13,682 bp from the start codon of the integrase gene fragment to the stop codon of a mobile element protein, of which the last seven nucleotides overlap with the end of the transcriptional regulator YcjW. This latter gene (part of the multi-gene Ycj putative regulator cluster) flanks the downstream end of the prophage, while upstream from the integrase fragment, the gene encoding peptide transport system permease SapC can be found, with a 294 nt long non-coding region between them. The PHASTER search originally gave a greater length, but it also included the YcjW gene itself, as well as a multi-gene operon encoding a peptide ABC transporter system and two IS*110* elements into the prophage sequence.

**FIGURE 2:**
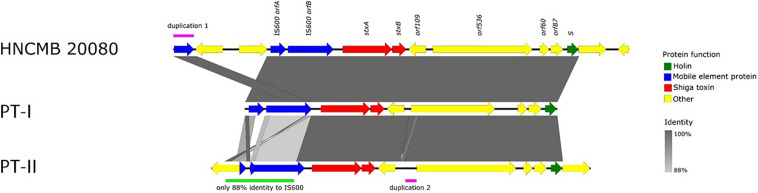
Schematic representation of the two types of Stx1-prophages present in the genomes of SD1 strains.

The prophage region is located in exactly the same region in all SD1 strains with complete genomes available, as well as in the 10 strains sequenced in Njamkepo et al. (2016), where PacBio reads were made (Table 1). The size and genetic content is highly similar in all cases, the sequence identity is above 99% throughout the whole region. The only exception is in strain Sd1617, where *orfA* of IS*600* upstream of the *stx* genes is truncated. Based on this difference, we termed the two main prophage types “I” and “II” (heretofore referred to as PT-I and PT-II, with PT standing for “prophage type”). The detailed representation of the Stx prophage region with the two main variants is shown on Figure 2. The list of SD1 strains with complete genomes and the type of their Stx prophages is given in Table 1.

### Antimicrobial Resistance

As the genome sequencing revealed that HNCMB 20080 harbors the gene *mdfA* associated with macrolide-resistance, we conducted a MIC test with erythromycin. The MIC of HNCMB 20080 against this antibiotic proved to be 64 μg/ml.

### Phage Induction

Phage induction of HNCMB 20080 was attempted with various agents (mitomycin C, norfloxacin, UV-light), but none of the experiments resulted in detectable lytic activity on *E. coli* K-12 derivative strain MG1655.

### Lysogenization Experiments

Lysogenization of HNCMB 20080 was attempted with wild type and modified Stx phages listed in Table 3. None of the experiments yielded lysogenic cell lines.

**TABLE 3:** Attempts for Lysogenization and Phage Transduction.

**Phage name**	**Stx type**	**Source strain**	**References**
3538Δstx2:cat	Knock-out (originally Stx2)	EHEC O157:H7 3538/95	Schmidt et al., 1999
933WΔstx2:gfp:cat	Knock-out (originally Stx2)	EHEC O157:H7 EDL933 (original 933W)	Imamovic et al., 2016
4979	Stx2	EHEC O157:H7 4979	Tóth et al., 2009
HUN-2013	Stx1	*Shigella sonnei* 75/02	Tóth et al., 2016

## Discussion

We sequenced and assembled the complete genome of *S. dysenteriae* strain HNCMB 20080, a historical strain from the 1950s isolated in Hungary. A draft genome of the strain was published earlier as part of a global *S. dysenteriae* sequencing project (Njamkepo et al., 2016). Albeit the project included a comprehensive representative collection of samples, complete genome sequences available for this pathogen are still relatively low in number (Table 1), representing only 3% (11/335) of all genome sequences available in GenBank after the study.

In general, the chromosomal structure of *S. dysenteriae* strains, including HNCMB 20080 is highly conserved, which is also supported by the fact that the complete and PacBio-sequenced genomes of SD1 known up to date are restricted to only two MLST types, except for the untypeable strain Sd1617 (Table 1). However, there were several chromosomal inversions, and all of them associated with insertion sequence (IS) elements. In the first *Shigella* sequencing project IS elements were highlighted as having a likely role in genomic rearrangements in *Shigella* genomes, with *S. dysenteriae* strain Sd197 having the most of these genomic features (Yang et al., 2005).

Phylogenetic relations for the available complete SD1 genomes could not be resolved adequately using MLST, as all strains except for ATCC13313 and 1617 belonged to the same sequence type. The core SNP-based phylogeny created with ParSNP showed that the lineages defined by Njamkepo et al. (2016) using the SNP-based method SMALT^2^ grouped together, but interestingly, out of the lineage III strains, HNCMB 20080 fell closest to the clusters containing lineage IV strains which harbored strains isolated in the 1980s and 1990s, unlike HNCMB 20080 which was isolated in the 1950s.

HNCMB 20080 was examined in detail because it is a) a historical strain and b) considered sensitive to all major antibiotic classes. The genomic investigation only revealed a macrolide resistance gene *mdfA*, and the strain showed a 64 μg/ml MIC against erythromycin, which is at the lower end of the reported range between 64 and 1,024 μg/ml for Enterobacteria (Greenberg et al., 1982; Boumghar-Bourtchai et al., 2008).

The genome of HNCMB 20080 contains 27 prophages or prophage-like regions altogether, including the one containing the Stx encoding gene cluster. However, probably due to the passages during storage throughout the decades since its isolation, the strain has most likely lost its pInv plasmid.

For Shiga toxin-producing *E. coli* (STEC), there have been extensive investigation in the past three decades regarding the genetic properties of Stx encoding genes and its genomic environment. It is generally accepted that it is carried by a lambdoid prophage, but in contrast to STEC, the exact genetic background and origin of Stx in the case of SD1 strains still remains unresolved. Since the work of Unkmeir and Schmidt (2000), there have been no further studies specifically targeting the Stx-carrying prophages of this pathogen.

In light of the above considerations, we examined the genetic structure and relatedness of Stx-encoding prophages found in complete SD1 genomes available in GenBank. We found that the region is remarkably conserved, spanning 13.5 kbp and integrated into the same site in every strain i.e., between the *sapC* and *ychK* genes (McDonough and Butterton, 1999). Two main sequence variants, PT-I and PT-II could be distinguished. Currently, the latter is represented by only one strain, Sd1617. In all cases, the prophage is defective, missing most of the key structural genes necessary for it to follow a lytic lifestyle.

The conserved nature of the phage indicated that its integration in the genome most likely precedes the isolation of the first strains, as even in the case of the historical strain M116, which was isolated between 1915 and 1918 (Njamkepo et al., 2016), the region does not differ from those found in the more recently isolated strains. However, as we have noted above, strains with complete genome only represent a small fraction of all the sequenced strains. Completion of further genomes could still reveal new, or possibly more complete variants of the Stx prophage in SD1 strains.

Current evidence also suggests that SD1 had acquired and domesticated the phage earlier than the isolation of the first strains at the turn of the nineteenth and twentieth centuries. This notion strongly suggests that STEC strains obtained Stx1 and Stx2 prophages much later than SD1 strains, as the first STEC were only identified in the 1980s (Riley et al., 1983). Moreover, Stx1-producing *S. flexneri* and *S. sonnei* strains were identified as late as in the twenty-first century (Gray et al., 2014; Carter et al., 2016). The notion of their recent acquisition of Stx1 phages is also supported by the inducibility of the phages (Strauch et al., 2001; Tóth et al., 2016). Although sequence data strongly suggests the common origin of the Stx prophages, their diverse states of detoriation and inducibility indicates that they were acquired at different timepoints throughout the past 100 years.

The genomic data of SD1 strains seem to exclude the possibility of the phage being transduced from them to STEC strains, albeit in the case of the prototypic enterohemorrhagic *E. coli* (EHEC) O157:H7 Sakai strain, the Stx1 gene is harbored on a defective prophage, which in turn can be mobilized through recombination with the Stx2-harboring inducible prophage (Asadulghani et al., 2009; Sváb et al., 2015). Such a recombination event could also have occurred between SD1 Stx1-carrying prophage and a more complete lambdoid phage. However, the observed resistance of HNCMB 20080 to infection with Stx2-carrying phages and its derivates, does not support this possibility. The results of our phage induction experiment seem to exclude the possible recombination, or the presence of satellite or helper phages (reviewed by Penadés and Christie, 2015) between the Stx1 prophage and other prophages in the genome of HNCMB 20080.

In summary, we determined the complete genome sequence of a historical SD1 isolate from Hungary, and the conserved nature of SD1 genomes, together with the presence of multiple chromosomal inversions was confirmed. We have found that the Stx1 prophage also has a conserved sequence, and established the existence of two variants throughout all complete SD1 genomes available so far. The prophage was found uninducible. For further exploration of the evolutionary history of Stx-carrying prophages, as well as that of their carriage in SD1 strains, a larger set of high-quality complete genomes, supplemented with traditional phage induction and infection experiments would be beneficial.

## Data Availability Statement

The datasets presented in this study can be found in online repositories. The names of the repository/repositories and accession number(s) can be found below: https://www.ncbi.nlm.nih.gov/genbank/, CP061527-CP061529; https://www.ncbi.nlm.nih.gov/, PRJNA662859.

## Author Contributions

IT and TC conceived and designed the study. DS and IT performed phenotypic experiments. BH and GM performed the Illumina sequencing and conducted the assembly. LF and JF conducted the Nanopore sequencing and hybrid assembly, as well as the assembly of PacBio sequences and other bioinformatics work. DS performed the additional comparative bioinformatics work and drafted the manuscript. All authors reviewed and wrote parts of the manuscript.

## Conflict of Interest

BH was employed by company Seqomics Ltd. The remaining authors declare that the research was conducted in the absence of any commercial or financial relationships that could be construed as a potential conflict of interest.

## Footnotes

1 https://github.com/rrwick/Porechop accessed on August 28th, 2020.

2 https://www.sanger.ac.uk/tool/smalt-0/accessed on November 26th, 2020.
